# Identification of novel candidate disease genes from de novo exonic copy number variants

**DOI:** 10.1186/s13073-017-0472-7

**Published:** 2017-09-21

**Authors:** Tomasz Gambin, Bo Yuan, Weimin Bi, Pengfei Liu, Jill A. Rosenfeld, Zeynep Coban-Akdemir, Amber N. Pursley, Sandesh C. S. Nagamani, Ronit Marom, Sailaja Golla, Lauren Dengle, Heather G. Petrie, Reuben Matalon, Lisa Emrick, Monica B. Proud, Diane Treadwell-Deering, Hsiao-Tuan Chao, Hannele Koillinen, Chester Brown, Nora Urraca, Roya Mostafavi, Saunder Bernes, Elizabeth R. Roeder, Kimberly M. Nugent, Patricia I. Bader, Gary Bellus, Michael Cummings, Hope Northrup, Myla Ashfaq, Rachel Westman, Robert Wildin, Anita E. Beck, LaDonna Immken, Lindsay Elton, Shaun Varghese, Edward Buchanan, Laurence Faivre, Mathilde Lefebvre, Christian P. Schaaf, Magdalena Walkiewicz, Yaping Yang, Sung-Hae L. Kang, Seema R. Lalani, Carlos A. Bacino, Arthur L. Beaudet, Amy M. Breman, Janice L. Smith, Sau Wai Cheung, James R. Lupski, Ankita Patel, Chad A. Shaw, Paweł Stankiewicz

**Affiliations:** 1 Department of Molecular and Human Genetics, Baylor College of Medicine, One Baylor Plaza, Houston, TX 77030-3411 USA; 2 Institute of Computer Science, Warsaw University of Technology, Warsaw, 00-665 Poland; 3 Department of Medical Genetics, Institute of Mother and Child, Warsaw, 01-211 Poland; 4 Baylor Genetics, Houston, TX 77021 USA; 5 Division of Pediatric Neurology, University of Texas Southwestern Medical Center, Dallas, TX 75390 USA; 6 Children’s Health Dallas, Dallas, TX 75235 USA; 7 Department of Pediatrics, University of Texas Medical Branch, Galveston, TX 77555 USA; 8 Department of Biochemistry and Molecular Biology, University of Texas Medical Branch, Galveston, TX 77555 USA; 9 Department of Pediatric, Section of Child Neurology, Baylor College of Medicine, Houston, TX 77030 USA; 10 Department of Psychiatry and Behavioral Sciences, Child and Adolescent Psychiatry Division, Baylor College of Medicine, Houston, TX 77030 USA; 11 Department of Pediatrics, Baylor College of Medicine, Houston, TX 77030 USA; 12 Jan and Dan Duncan Neurological Research Institute, Texas Children’s Hospital, Houston, TX 77030 USA; 13 Department of Clinical Genetics, Helsinki University Hospital, Helsinki, 00029 Finland; 14 Genetics Division, Department of Pediatrics, University of Tennessee Health Science Center, Memphis, TN 38105 USA; 15 Le Bonheur Children’s Hospital, Memphis, TN 38103 USA; 16 Phoenix Children’s Hospital, Phoenix, AZ 85016 USA; 17 Department of Pediatrics, Baylor College of Medicine, San Antonio, TX 78207 USA; 18 Northeast Indiana Genetic Counseling Center, Wayne, IN 46804 USA; 19 Section of Clinical Genetics & Metabolism, Department of Pediatrics, University of Colorado School of Medicine, Aurora, CO 80045 USA; 20 Department of Psychiatry Erie County Medical Center, Buffalo, NY 14215 USA; 21 Division of Medical Genetics, Department of Pediatrics, McGovern Medical School, The University of Texas Health Science Center at Houston, Houston, TX 77030 USA; 22 St. Luke’s Children’s Hospital, Boise, ID 83702 USA; 23 The National Human Genome Research Institute, Bethesda, MD 20892 USA; 24 Seattle Children’s Hospital, Seattle, WA 98105 USA; 25 Department of Pediatrics, Division of Genetic Medicine, University of Washington, Seattle, WA 98195 USA; 26 Dell Children’s Medical Center, Austin, TX 78723 USA; 27 Child Neurology Consultants of Austin, Austin, TX 78731 USA; 28 THINK Neurology for Kids/Children’s Memorial Hermann Hospital, The Woodlands, TX 77380 USA; 29 Division of Plastic Surgery, Baylor College of Medicine, Houston, TX 77030 USA; 30 Centre de Génétique et Centre de Référence Anomalies du Développement et Syndromes Malformatifs de l’Est, FHU-TRANSLAD, CHU Dijon, Dijon, France; 31 Texas Children’s Hospital, Houston, TX 77030 USA

**Keywords:** Exon targeted array CGH, Intragenic copy number variants, CNVs, de novo variants

## Abstract

**Background:**

Exon-targeted microarrays can detect small (<1000 bp) intragenic copy number variants (CNVs), including those that affect only a single exon. This genome-wide high-sensitivity approach increases the molecular diagnosis for conditions with known disease-associated genes, enables better genotype–phenotype correlations, and facilitates variant allele detection allowing novel disease gene discovery.

**Methods:**

We retrospectively analyzed data from 63,127 patients referred for clinical chromosomal microarray analysis (CMA) at Baylor Genetics laboratories, including 46,755 individuals tested using exon-targeted arrays, from 2007 to 2017. Small CNVs harboring a single gene or two to five non-disease-associated genes were identified; the genes involved were evaluated for a potential disease association.

**Results:**

In this clinical population, among rare CNVs involving any single gene reported in 7200 patients (11%), we identified 145 de novo autosomal CNVs (117 losses and 28 intragenic gains), 257 X-linked deletion CNVs in males, and 1049 inherited autosomal CNVs (878 losses and 171 intragenic gains); 111 known disease genes were potentially disrupted by de novo autosomal or X-linked (in males) single-gene CNVs. Ninety-one genes, either recently proposed as candidate disease genes or not yet associated with diseases, were disrupted by 147 single-gene CNVs, including 37 de novo deletions and ten de novo intragenic duplications on autosomes and 100 X-linked CNVs in males. Clinical features in individuals with de novo or X-linked CNVs encompassing at most five genes (224 bp to 1.6 Mb in size) were compared to those in individuals with larger-sized deletions (up to 5 Mb in size) in the internal CMA database or loss-of-function single nucleotide variants (SNVs) detected by clinical or research whole-exome sequencing (WES). This enabled the identification of recently published genes (*BPTF*, *NONO*, *PSMD12*, *TANGO2*, and *TRIP12*), novel candidate disease genes (*ARGLU1* and *STK3*), and further confirmation of disease association for two recently proposed disease genes (*MEIS2* and *PTCHD1*). Notably, exon-targeted CMA detected several pathogenic single-exon CNVs missed by clinical WES analyses.

**Conclusions:**

Together, these data document the efficacy of exon-targeted CMA for detection of genic and exonic CNVs, complementing and extending WES in clinical diagnostics, and the potential for discovery of novel disease genes by genome-wide assay.

**Electronic supplementary material:**

The online version of this article (doi:10.1186/s13073-017-0472-7) contains supplementary material, which is available to authorized users.

## Background

Clinical application of genome-wide assay by chromosomal microarray analysis (CMA) has significantly improved the detection rate for molecular diagnoses in clinical genomics diagnostics [1], enabling the elucidation of pathogenic copy number variants (CNVs) in individuals with various conditions, including congenital anomalies, intellectual disability/developmental delay (ID/DD), autism spectrum disorder (ASD), epilepsy, heart defects, and neuropsychiatric diseases [2–11]. CNVs smaller than 400 kb in size are challenging for clinical interpretation and, when not involving known genes [2, 12, 13], they are often not reported in routine clinical CMA. Nevertheless, such CNVs have been documented to contribute to cognitive phenotypes in population studies [14, 15].

Whereas the positive correlation between the size of a CNV and likelihood of pathogenicity guided the size cutoffs used for the clinical reporting of CNVs [5, 13], gene content is also an important factor in the determination of potential CNV pathogenicity. Disease-causing CNVs may be as small as a single exon [16–24], which still remain beyond the detection limits of whole-exome sequencing (WES) [25]. To improve the detection rate for such pathogenic CNVs, several groups developed exon-focused arrays with a sufficient number of interrogating oligo probes to target single exons of both known disease-associated genes and developmentally important genes that are not yet associated with human disease [12, 13, 26–33].

In 2010, we reported the CMA results in 3743 patients using our first version of a clinical exon-targeted array (OLIGO V8) [29]. We demonstrated that increasing array resolution to single exons not only allowed detection of small CNVs in the known disease genes, but also provided new opportunities for novel gene discoveries and the ability to detect somatic mosaicism for intragenic CNVs [29, 34].

Despite the advances in molecular diagnostics using genome-wide assays [1], WES and targeted next-generation sequencing (NGS) studies have limited ability to detect small intragenic CNVs [25] and therefore cannot currently reveal the totality of disease genes and pathogenic alleles. To further investigate this hypothesis and to assess the efficacy of exon-targeted CMA to identify putative novel disease genes, we queried our database of exon-targeted clinical CMA performed in 63,127 patients at Baylor Genetics (BG) Laboratories, with a particular focus on small de novo autosomal and X-linked (in males) CNVs involving genes that are currently not associated with human disease. These variants have increased likelihood to be pathogenic and may lead to the identification of the novel candidate disease genes [3, 23, 27, 35, 36]. We also cross-referenced these “gene level” data with single nucleotide variants (SNV) in the same gene detected in the clinical WES laboratory at BG and research exomes from the Baylor Hopkins Center for Mendelian Genomics (BHCMG).

Previous works demonstrating increased performance of exon-targeted CGH arrays focused mostly on the improvements in detection of CNVs involving known disease genes. Our results show that the systematic study of the large clinical cohort using exon-targeted CMA can be successfully used to discover novel disease genes, especially utilizing CNVs involving the candidate or potential new disease genes and with further integration of SNV data from WES.

## Methods

### Microarrays

In 2008, we designed and clinically implemented array comparative genomic hybridization (aCGH) with exonic coverage of over 1700 disease and candidate disease genes (OLIGO V8) and demonstrated its efficacy for detection of pathogenic exonic losses or gains as small as a few hundred base pairs in size [29]. This work suggested that as many as 10% (3/30 in known disease genes) of small intragenic CNVs might represent mosaic mutant alleles. Subsequently, we expanded our custom-designed oligo array to include > 4800 genes, including autosomal recessive disease genes (OLIGO V9 [37], V10, and V11).

We retrospectively analyzed CMA data from 63,127 patients (most diagnosed with neurodevelopmental defects) referred for CMA between April 15, 2007 and February 17, 2017 using six different versions of customized oligonucleotide arrays (OLIGO V6-V11; Agilent Technologies Inc., Santa Clara, CA, USA) developed at BG Laboratories. Among 63,127 patients, 46,755 were analyzed using microarrays targeting 1700 genes (OLIGO V8) or the subsequent microarray versions targeting > 4800 genes (OLIGO V9, V10, and V11) [12, 29]. In these microarrays, more than 90% of the exons in targeted genes were covered with at least three interrogating oligonucleotides, with an average of > 4.2 probes per exon, whereas intronic probes were uniformly distributed every 10 kb. In addition, for the purposes of normalization and statistical analyses of raw data, the design included unique sequence interrogating oligonucleotide probes for the entire genome covered at an average resolution of 30 kb (excluding segmental duplications). The procedures for DNA digestion, labeling, and hybridization for the oligo arrays were performed according to the manufacturers’ instructions, with minor modifications [38–40].

We used an in-house developed software to detect CNVs from aCGH data. The algorithm requires at least three consecutive probes with a log2 ratio < – 0.6 to detect deletion or at least three consecutive probes with a log2 ratio > 0.4 to detect duplication. CNVs < 500 kb with no RefSeq genes within the intervals or CNVs within segmental duplication (SDs) and CNVs in benign polymorphic regions, such as those listed in Database of Genomic Variants (DGV) are generally not reported. CNVs located in the regions known to be disease-associated and likely exhibiting incomplete penetrance, variable expressivity, or representing a potential recessive carrier state can be reported despite their overlap with DGV CNVs. The remaining CNVs are then classified as: (1) pathogenic = pathogenic CNVs includes aneuploidy, known microdeletion/microduplication, deletion, and intragenic duplication genomic intervals involving known dosage-sensitive autosomal-dominant (AD) disease genes, or deletion of any size involving a dosage sensitive gene associated with AD, haploinsufficiency, or other likely pathogenic consequence. Moreover large duplications (> 2 Mb) with genes or duplications > 1 Mb with genes known to be dosage-sensitive are also classified as pathogenic CNVs; (2) likely benign = < 1 Mb CNVs with no genes in the intervals or CNVs in the DGV database and have been reported to be inherited multiple times in our CMA database; (3) loss/gain in non-disease region = rare CNVs < 1 Mb with genes not implicated in disease phenotypes; (4) loss/gain of uncertain clinical significance = CNVs that have genes, but there is no supporting evidence of pathogenicity. CNVs of uncertain significance are usually investigated by parental studies. CNVs that have been shown to exhibit incomplete or uncertain penetrance were classified as such.

### Whole-exome sequencing data

WES was performed in ~ 9000 individuals sequenced at BG and in the cohort of ~ 6000 samples sequenced in the Human Genome Sequencing Center (HGSC) at BCM through the BHCMG research initiative [41] as described previously [23, 42–44].

### Computational parsing of clinical CMA database

The main aim of our retrospective analyses was to identify potential pathogenic or likely pathogenic CNVs affecting genes that thus far were not disease-associated. Subsequently, we queried the BG CMA database for other overlapping submicroscopic CNV deletions up to 5 Mb in size (de novo, inherited, or of unknown parental origin) that encompassed at least one gene that we consider as novel or recently published candidate disease gene.

Genes affected by single-gene CNVs and those included in de novo or hemizygous CNVs affecting 2–5 genes were classified according to their disease status using the lists of known AD, autosomal-recessive (AR), or X-linked (XL) disease genes, as defined in the Online Mendelian Inheritance in Man (OMIM) database (http://omim.org/) [29, 45, 46]. For each gene, we performed an extensive literature search for genotype–phenotype correlations. Moreover, all genes were cross-referenced with the Simons Foundation Autism Research Initiative (SFARI) database (https://sfari.org/) of ASD-related genes [47].

In addition, we searched the BG and BHCMG exome databases for predicted loss-of-function (LOF) (i.e. stop-gain, frameshift, or splicing) variants in the candidate genes to potentially provide further evidence of their disease-association. From the initial set, we excluded variants with a total coverage of < 20 reads or the ratio of variant to total reads below 0.2. We also removed variants with minor allele frequency (MAF) > 0.001 in ESP [48], 1000 Genomes [49], or our local exome databases or with MAF > 0.0001 in ExAC (Exome Aggregation Consortium, http://exac.broadinstitute.org) [50].

To assess the predicted probability of exhibiting haploinsufficiency for a given gene, we used haploinsufficiency scores calculated by Huang et al. [51]. These predictions were generated using classification models trained on known haploinsufficient genes and genes disrupted by unambiguous LOF variants in at least two apparently healthy individuals. Genes with high rank scores (0–10%) indicate that the gene is likely to exhibit haploinsufficiency. In our evaluation of a particular gene, we also consider the pLI score, i.e. the probability that the gene is intolerant to LOF variants. This score was generated based on the analysis of the ratio of the number of observed vs. expected LOF variants in the ExAC population [50, 52]. Genes with high pLI scores (pLI ≥ 0.9) are extremely LOF-intolerant, whereby genes with low pLI scores (pLI ≤ 0.1) are LOF-tolerant.

### Fluorescent in situ hybridization (FISH)

FISH analyses were performed with bacterial artificial chromosome (BAC) or fosmid clones using standard procedures [53].

### Breakpoint junction sequencing

We designed a customized high-density CGH array (HD-aCGH, AMADID 081888) for analyzing CNVs detected by CMA to further resolve breakpoint junctions and determine the nucleotide sequence of selected breakpoints. Long-range polymerase chain reaction (PCR) followed by Sanger sequencing of the PCR products was performed, as described, to obtain base-pair resolution of the breakpoint junctions [54]. Breakpoint junction sequencing was performed for small deletions in which the involved exons could not be determined due to a limited resolution of our clinical array.

### Parental studies

The inheritance status of CNVs was determined by analyzing parental DNA using CMA or FISH. Note that in the case of de novo events, we did not formally confirm paternity and maternity by molecular testing.

## Results

**Fig. 1:**
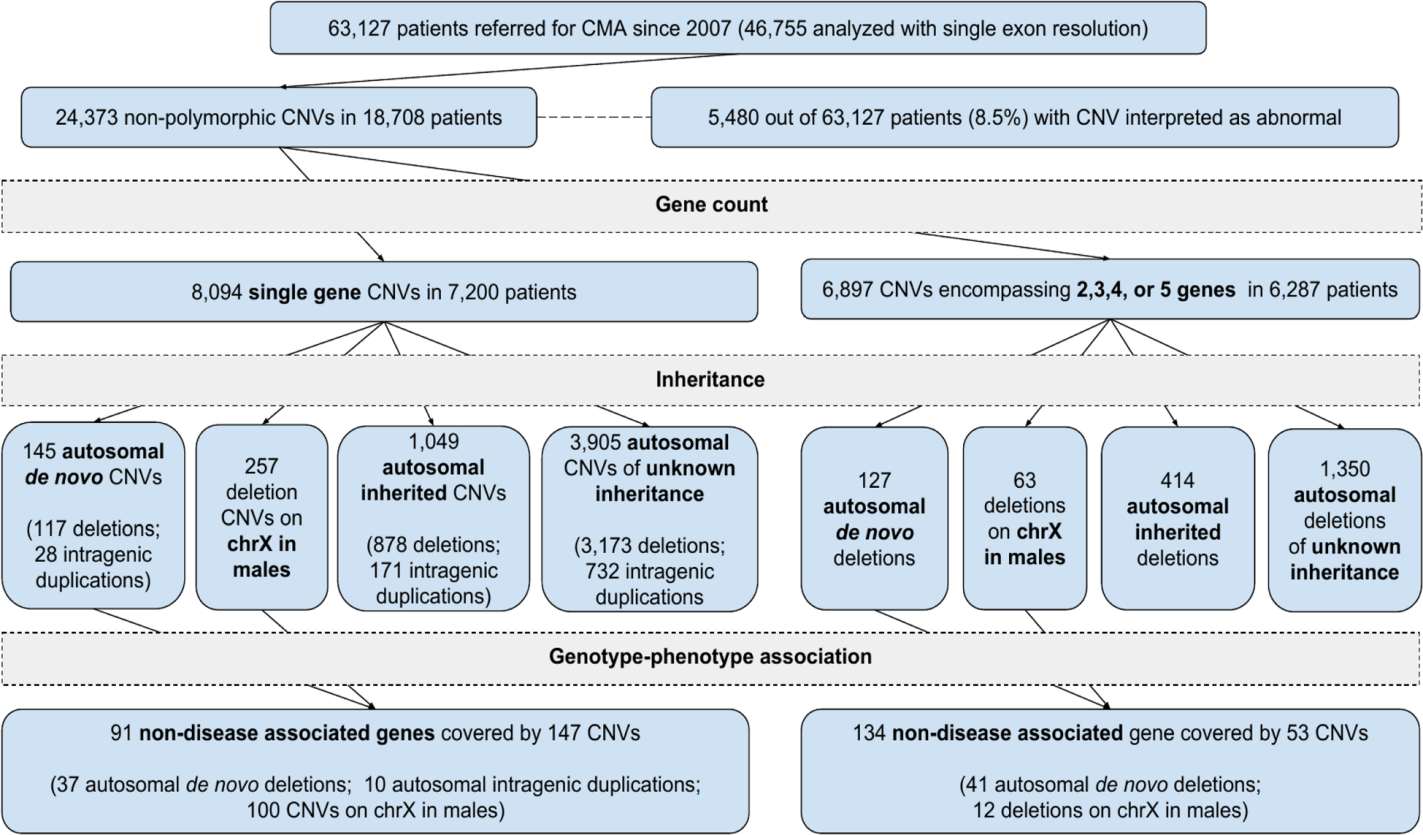
Overview of the CNV filtering strategy

Out of 24,373 non-polymorphic CNVs detected in 18,708 patients, genome-wide clinical aCGH studies identified 7200 individuals with 8094 CNVs involving a single gene, including 145 de novo autosomal (117 losses and 28 intragenic gains) and 257 X-linked (in males) deletion CNVs, and 1049 inherited CNVs (878 losses and 171 intragenic gains) on autosomes (Fig. 1). Sizes of CNVs overlapping a single gene were in the range of ~ 100 bp to ~ 5.8 Mb with a median size of 94 kb. Importantly, 1857 (23%) of single gene CNVs affected only a single exon. We also found 6287 individuals with 6897 CNVs encompassing 2–5 genes (including disease-associated and non-disease-associated genes), including 127 de novo autosomal, 63 X-linked (in males), and 414 inherited autosomal CNV deletions (Fig. 1).

### Most common single-gene CNVs affect primarily known disease genes

In our clinical cohort, the most common single-gene CNVs include *CHRNA7* (OMIM* 118511) (14 deletions/312 duplications), *IMMP2L* (OMIM* 605977) (151 deletions/1 duplication), *TMLHE* (OMIM* 300777) (125 deletions/21 duplications), *RBFOX1* (OMIM* 605104) (60 deletions/62 duplications), *DMD* (OMIM* 300377) (74 deletions/32 duplications), *NRXN1* (OMIM* 600565) (90 deletions/9 duplications), *CNTN6* (OMIM* 607220) (22 deletions/63 duplications), and *PARK2* (OMIM* 602544) (50 deletions/17 duplications). Of those, events in *CHRNA7*, *DMD*, and *NRXN1* [34] were interpreted as directly causative for the patients’ phenotypes, whereas CNVs involving *TMLHE* [32], *PARK2* [55], and *RBFOX1* [56, 57] may confer susceptibility to disease or represent an allele for a recessive carrier state [58]. In total, we identified 111 known disease genes that were potentially disrupted by de novo autosomal or X-linked (in males) single-gene CNVs.

### Recently proposed and potential novel disease genes

We identified 91 genes that were either recently proposed to be candidate disease-associated or non-disease-associated genes. These were disrupted by 37 de novo autosomal single-gene deletions, 100 X-linked CNVs (97 deletions and three intragenic duplications in males), and ten de novo intragenic duplications on autosomes (see Fig. 1 and Additional file 1).

To search for additional candidate disease genes, we extended our analyses to 6897 CNV deletions harboring 2–5 non-disease genes. We identified 134 distinct recently proposed or not yet disease-associated genes involved in 41 de novo autosomal and 12 X-linked (in males) deletions (see Fig. 1 and Additional file 2).

To further narrow the list of candidate disease-causing genes, we considered the following factors: (1) the number of de novo CNVs determined for each gene (Additional files 1 and 2); (2) additional CNVs < 5 Mb in size found in our cohort; (3) LOF variants found in ~ 15,000 WES cases (from BG and BHCMG “disease cohorts”); (4) phenotypic overlap among patients; (5) literature records supporting disease association; (6) predictions of haploinsufficiency [51] and intolerance to LOF [50] of the identified variants. Using these criteria, we found evidence supporting the contention of recently published disease genes, including *BPTF* (OMIM* 601819) [59], *NONO* (OMIM* 300084) [60], *PSMD12* (OMIM* 604450) [61], *TANGO2* (OMIM* 616830) [62, 63], *TRIP12* (OMIM* 604506) [64, 65], and likely *MAGED1* (OMIM* 300224) [66]. Furthermore, we found genes recently reported as disease-associated, including *TBR1* (OMIM* 604616) [67] and *CLTCL1* (OMIM* 601273) [68], as well as genes not yet associated with diseases.

**Fig. 2:**
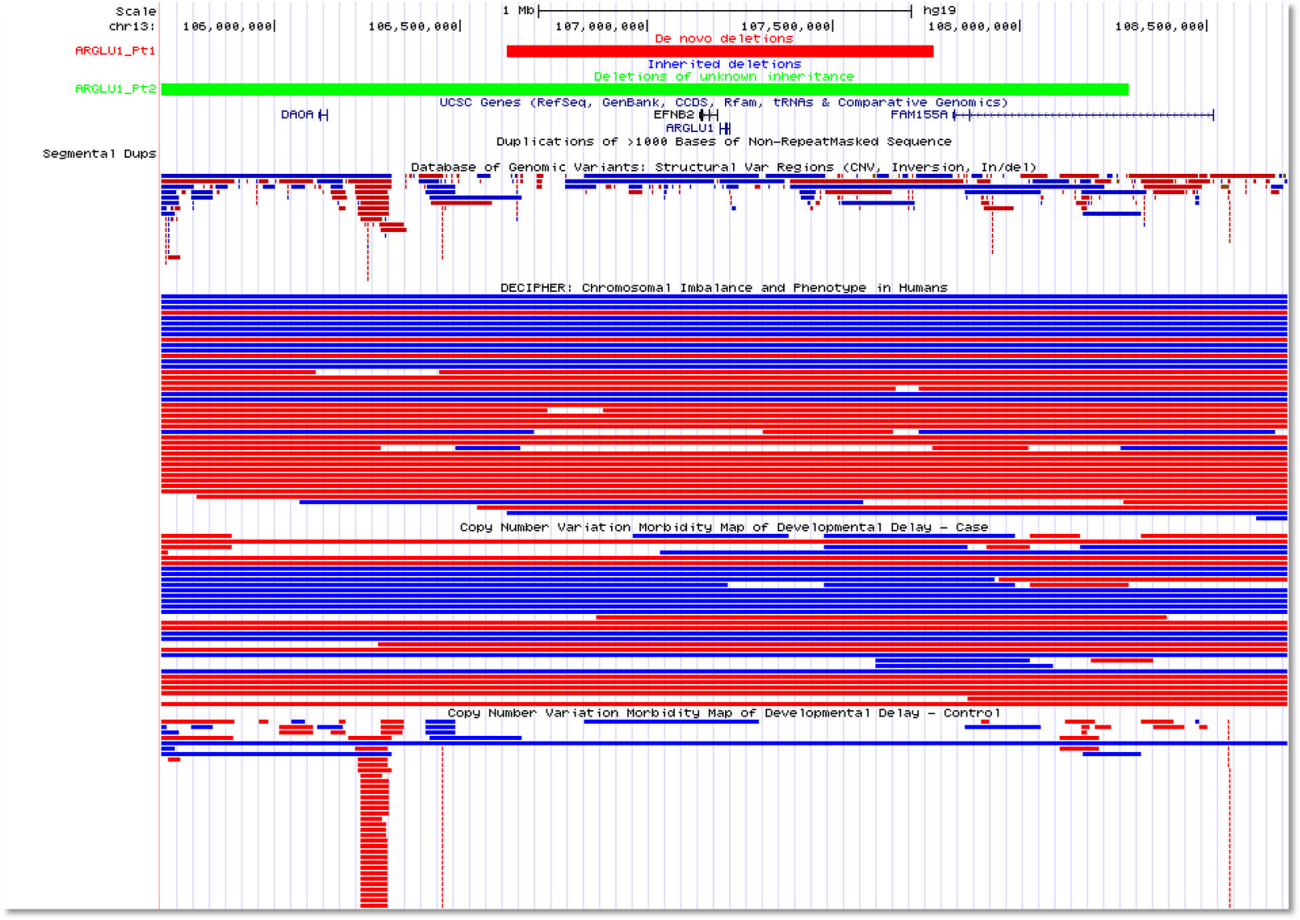
CNVs in *ARGLU1/EFNB2*, including de novo (red) and deletions of unknown inheritance (green)

**Fig. 3:**
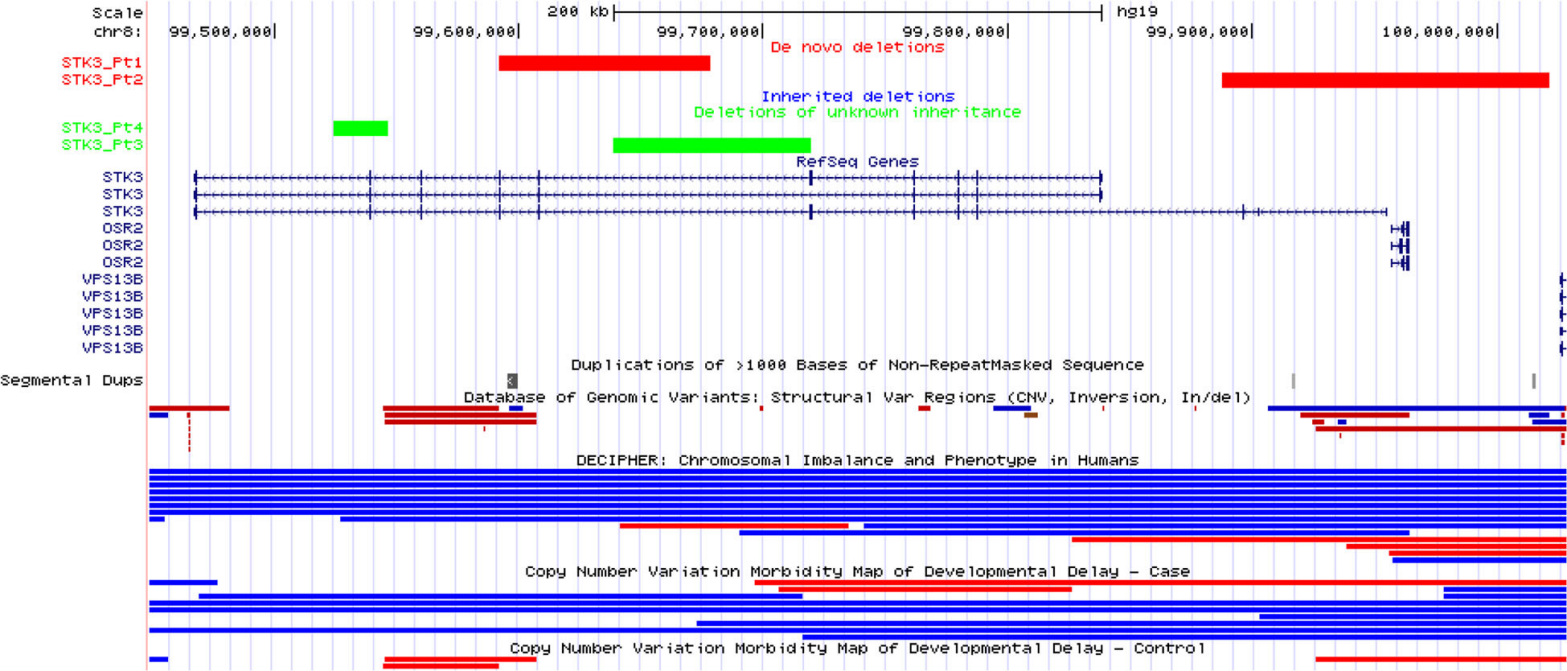
CNVs in *STK3*, including de novo (red) and deletions of unknown inheritance (green)

**Fig. 4:**
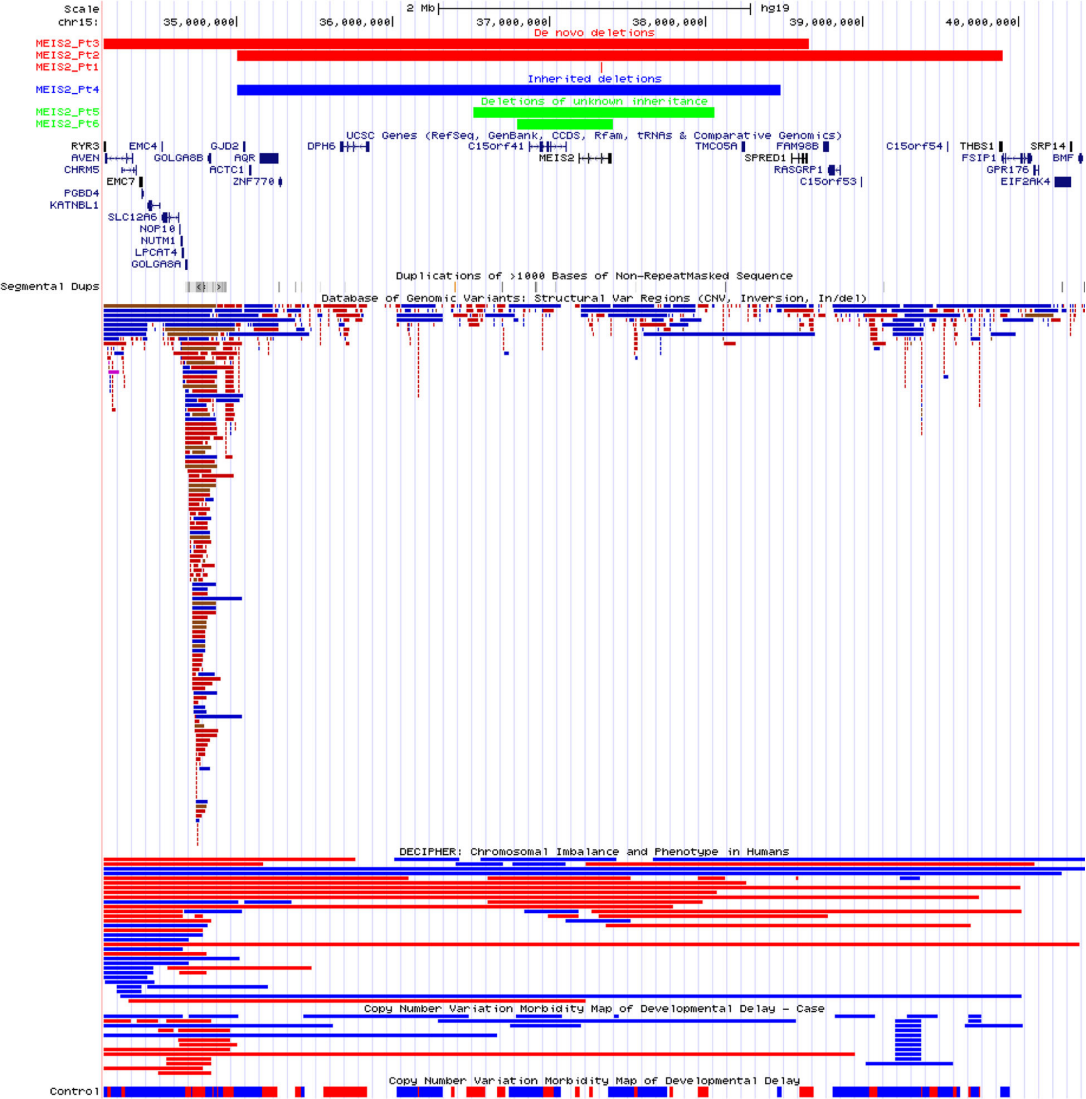
CNVs in *MEIS2*, including de novo (red), inherited (blue), and deletions of unknown inheritance (green)

**Fig. 5:**
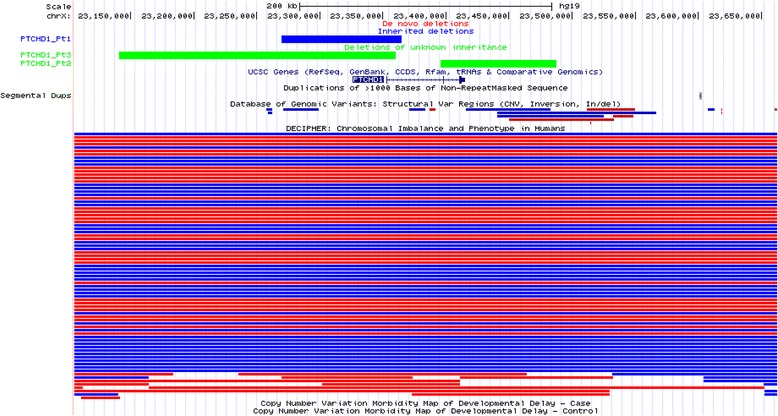
CNVs in *PTCHD1*, including inherited (blue) and deletions of unknown inheritance (green)

We selected two novel non-disease-associated genes, *STK3* (OMIM* 605030) mapping to 8q22.2 and *ARGLU1* (OMIM* 614046) that maps in 13q33 for further molecular and clinical analyses, to determine whether these could be novel disease genes. In addition, we attempted to expand the genotype–phenotype correlations for two recently proposed candidate disease genes, *MEIS2* (OMIM* 601740) on 15q14 [69–74], and *PTCHD1* (OMIM* 3008280) on Xp22.11 [75–80]. In total, we found 17 small CNV deletions (3.2 kb to 4.9 Mb in size with three CNVs < 50 kb) overlapping these four candidate disease genes, including eight de novo events (Figs. 2, 3, 4 and 5). However, paternity was not tested formally (i.e. by molecular markers) and thus non-paternity could not be ruled out for most of the cases. The investigation of ~ 15,000 WES samples from BG and BHCMG revealed rare LOF variants, providing additional support for potential genotype-phenotype correlations (Tables 1, 2, 3 and 4, patients with variants in *ARGLU1*, *STK3*, *MEIS2*, and *PTCHD1*, respectively; see also Additional file 3 for discussion on two other candidate disease genes, *AGBL4* (OMIM* 616476) and *CSMD1* (OMIM* 608397); Additional files 4, 5 and 6 for information on patients with additional variants in *ARGLU1/EFNB2*, *AGBL4*, and *CSMD1,* respectively; and Additional files 7 and 8 for visualization of CNVs in *AGBL4* and *CSMD1*, respectively).

**Table 1:** Clinical information on patients with *ARGLU1* and *EFNB2* variants

Case number	Pt1	Pt2	Pt3	Pt4 (DECIPHER 280488)
Sex	Male	Female	Male	Female
Variant	chr13:106,624,717-107,768,458 1.1 Mb del	chr13:104,114,620-108,292,078 4.1 Mb del	chr13:107211843delA; NM_018011:c.509delA; p.K170fs frameshift	chr13:106,884,343-110,711,191 3.8 Mb del
Confirmation method	FISH	N/A	PCR + Sanger	FISH
Affected genes	***ARGLU1***, ***EFNB2***	*DAOA*, *FAM155A*, ***ARGLU1***, ***EFNB2***	***ARGLU1***	*ABHD13*, ***ARGLU1***, ***EFNB2***, *FAM155A*, *IRS2*, *LIG4*, *MYO6*, *TNFSF13B*
Inheritance	De novo	Unknown	De novo	De novo
Parental studies	FISH	N/A	PCR + Sanger	FISH
Developmental delay/intellectual disability	**+**	**+**	**+**	**+**
Developmental regression	**+**	N/A	N/A	N/A
Autistic spectrum	+	N/A	N/A	N/A
Abnormal movement	N/A	N/A	+	N/A
Cerebellar hypoplasia	N/A	N/A	+	N/A
Oculomotor apraxia	N/A	N/A	+	N/A
Seizures/epilepsy	N/A	+	N/A	N/A
Other	N/A	Horse shoe kidney/ectopic kidney, dysmorphic features	N/A	N/A

*N/A* not available

Candidate genes are indicated in bold

**Table 2:** Clinical information on patients with *STK3* variants

Case number	Pt1	Pt2	Pt3	Pt4
Sex	Female	Male	Female	Male
Variant	chr8: 99,591,666-99,678,567 87 kb, exons 7-8 del	chr8: 99,883,084-100,026,306^a^ 143 kb, exons 1-3 del	chr8: 99,638,463-99,719,599 81 kb, exons 5-6 del	chr8:99,524,409-99,546,574^a^ 22 kb, exon 10 del
Confirmation method	FISH	FISH, PCR + Sanger	High-density CGH array	PCR + Sanger
Affected genes	*STK3*	*STK3*, *OSR2*	*STK3*	*STK3*
Inheritance	De novo	De novo	Unknown	Unknown
Parental studies	FISH	FISH	N/A	N/A
Developmental delay/intellectual disability	N/A	+	N/A	+
Multiple congenital anomalies	**+**	N/A	Skull, face, and neck anomalies	N/A
Other	Dysmorphic features	Radioulnar synostosis, hypospadias	N/A	Failure to thrive, hypotonia

^a^CNV coordinates obtained using PCR and Sanger sequencing of breakpoint junction

*N/A* not available

**Table 3:** Clinical information on patients with *MEIS2* variants

Reference	Previous cases							
Case number	Erdogan et al.; Chen et al.; Crowley et al.; Johansson et al. (5 cases in 2 families); Louw et al. (4 cases); Fujita et al.	Pt1	Pt2	Pt3	Pt4	Pt5	Pt6	Pt7 (DECIPHER 286841)
Gender	8 females; 6 males	Male	Male	Female	Male	Male	Male	Female
Variant	6 CNV del (123 kb – 5.6 Mb); 1 CNV dup (58 kb); 1 stop-gain; 1 frameshift	chr15: 37,328,986-37,332,249^a^	chr15: 35,001,138-39,899,594	chr15: 33,894,032-38,659,166	chr15: 35,001,138-38,474,933	chr15: 36,512,757-38,052,959^a^	chr15: 36,790,702-37,404,359^a^	chr15:36,606,006-37,515,525
		3.2 kb del	4.9 Mb del	4.8 Mb del	3.47 Mb del	1.54 Mb del	0.6 Mb del	0.9 Mb del
Confirmation method		PCR + Sanger	FISH	FISH	FISH	PCR + Sanger	PCR + Sanger	Unknown
Affected genes		*MEIS2*	*MEIS2* + 14 other genes	*MEIS2* + 21 other genes	*MEIS2* + 7 other genes	*MEIS2* + 1 other gene	*MEIS2* + 1 other gene	*MEIS2*
Inheritance	9 de novo; 4 inherited; 1 mosaic	De novo	De novo	De novo	Mat	Unknown	Unknown	De novo
Parental studies		CMA	FISH	FISH	FISH	N/A	N/A	Unknown
Cleft lip and cleft palate	12 out of 14	+	N/A	N/A	N/A	Bifid uvula	N/A	Bifid uvula
Cardiac malformation	Ventricular septal defect (7); atrial septal defect (2); LVOTO; CoA	NR	N/A	N/A	NR	NR	NR	Learning problems, aggressive behavior
Cognitive and behavioral phenotype	ID (7); delayed (6); ASD (2); LD (2)	NR	N/A	N/A	ASD	Global DD, ASD, ADHD	ASD?	Slower verbal development
Verbal developmental delay	4 out of 4	Possibly	N/A	N/A	NR	Delayed verbal milestones	Delayed language skills	NR
Motor developmental delay	12 out of 12	NR	N/A	N/A	NR	Delayed motor milestones	NR	NR
Walked at age	14 months – 3 years	NR	N/A	N/A	NR	2 years	NR	NR
Gastro-esophageal reflux	2 out of 2	NR	N/A	N/A	NR	NR	NR	NR
Other features	Hypotonia; prolapse of epiglottis; bilateral moderate hearing loss; agenesis of the right tympanic membrane; a gracile corpus callosum; congenital lobar emphysema, syndactyly; severe hypermetropia, severe constipation	NR	Hypertonia	MCA	NR	Asthma; sister with Ebstein’s cardiac anomaly; family history of ID; maternal prenatal cocaine use	NR	Calcaneovalgus, velopharyngeal insufficiency, asymmetric chest

^a^CNV coordinates obtained using PCR and Sanger sequencing of breakpoint junction; note that Sanger sequencing of breakpoint junction PCR amplification product in Pt1 was affected by the homonucleotide tracts (poly-A/T) close by the breakpoint junction; therefore, the CNV coordinates in Pt1 were determined by the coordinates of the poly A/T tracts

*N/A* not available, *NR* not reported, *ID* intellectual disability, *ASD* Autism Spectrum Disorder, *LD* learning disability, *LVOTO* left ventricular outflow tract obstruction, *CoA* coarctation of the aorta, *CHD* coronary heart disease, *MCA* multiple congenital anomalies, *Mat* maternal

**Table 4:** Clinical information on patients with PTCHD1 variants

Clinical features	Pt1	Pt2	Pt3
Gender	Male	Male	Male
Variant	chrX: 23,269,452-23,364,920	chrX: 23,395,713-23,487,393^a^	chrX: 23,140,737-23,360,470^a^
	95 kb, exon 1 deletion	92 kb, exons 2–3 deletion	220 kb, exon 1 deletion
Confirmation method	N/A	PCR + Sanger	PCR + Sanger
Affected genes	*PTCHD1*	*PTCHD1*	*PTCHD1*
Inheritance	Mat	Unknown	Unknown
Parental studies	CMA	N/A	N/A
ASD	+	N/A	Autistic features
DD/ID	+	+	+
ADHD	+	N/A	+
Other	Hypotonia, speech impairment	N/A	N/A

^a^ - CNV coordinates obtained using PCR and Sanger sequencing of breakpoint junction

*N/A* not available, *DD* developmental delay, *ID* intellectual disability, *Mat* maternal

### ARGLU1 *and* EFNB2 *that map to 13q33 as potential new disease genes*

In the BG CMA database, we found two CNVs involving *ARGLU1* and *EFNB2* (OMIM* 600527), including one ~ 1.1 Mb de novo deletion encompassing *ARGLU1* and *EFNB2* and one ~ 4.2 Mb deletion of unknown inheritance harboring *ARGLU1*, *DAOA*, *FAM155A*, and *EFNB2* (Table 1). In addition, one de novo ~ 3.8 Mb deletion encompassing *ARGLU1* and *EFNB2* was found in one DECIPHER (https://decipher.sanger.ac.uk/) patient, 280488. Moreover, in the BG WES database, we identified one de novo frameshift variant (g.13:107211843delA; NM_018011:c.509delA; p.K170fs) in *ARGLU1*. Our further investigation of five novel or very rare missense variants: one in *ARGLU1* (c.350G > A p.R117Q) and four in *EFNB2* (c.498A > C, p.Q166H; c.503C > T, p.A168V; c.796A > G, p.T266A; c.803C > T, p.S268L) revealed that these five variants were all inherited (Additional file 4). Importantly, putative LOF variants in *ARGLU1* and *EFNB2* have been rarely seen in the BG and BHCMG databases (one variant mentioned above for *ARGLU1* and zero for *EFNB2*), indicating their intolerance to haploinsufficiency. Prediction algorithms indicate that both *ARGLU1* and *EFNB2* are sensitive to LOF with haploinsufficiency scores of 5.27 and 1.35 and the probabilities of intolerance to LOF mutations (pLI scores) of 0.99 and 0.94, respectively.

### STK3 *may be associated with human disease phenotypes*

We identified four different-sized CNV deletions involving *STK3* (Table 2). Two of these deletions (87 kb and 143 kb in size) were confirmed to be de novo; inheritance of two other CNVs (81 kb and 22 kb in size) could not be determined. In the DECIPHER database, one patient (258095) had an intragenic deletion of exons 5 and 6 of unknown parental origin. Although computationally determined haploinsufficiency and pLI scores for *STK3* (0.12 and 0, respectively) do not favor a haploinsufficiency pathomechanism for this gene, the identification of de novo variants suggest a potential disease association.

### *Novel* MEIS2 *and* PTCHD1 *variants*

We identified new variants in *MEIS2* and *PTCHD1*, which have been recently recognized as disease-associated genes. We found six different-sized deletions involving *MEIS2* (Table 3). Three of them, including one small (~ 3.2 kb) and two large (~ 4.8 Mb and ~ 4.9 Mb) deletions were confirmed to be de novo; one deletion ~ 3.47 Mb was maternally inherited and the inheritance of two other (~ 1.54 Mb and ~ 0.6 Mb) CNVs remains unknown. Moreover, an ~ 909 kb de novo deletion encompassing *MEIS2* and three other genes was found in the DECIPHER database (patient 286841). Prediction algorithms indicate *MEIS2* as sensitive to LOF with a haploinsufficiency score of 0.68 and the pLI score of 0.99.

We found three male patients with hemizygous deletions varying in size between 92 kb and 220 kb and encompassing exon 1 (two cases) or exons 2 and 3 of the three-exon *PTCHD1* gene on Xp22.11 (Table 4). In the DECIPHER database, there are at least two males with an inherited *PTCHD1* deletion. pLI score of 0.95 strongly suggest that *PTCHD1* is intolerant to LOF variants.

## Discussion

Clinical WES studies showed that pathogenic variants occur de novo in ~ 87% of patients with an established molecular diagnosis for an autosomal dominant disease trait [42]. Moreover, de novo mutations, both SNV and CNV, have been demonstrated to represent an important cause of ID/DD [81, 82] and damaging de novo mutations are significantly enriched (*P* = 8.0 × 10^-9^; odds ratio [OR] = 1.84) in patients with ASD when compared to controls [83]. CNV alleles, whether intragenic or gene-encompassing, represent a key modality of disease causing variation as heterozygous alleles associated with dominant disease traits or contributing to carrier state for recessive traits [58]. Genomic CNV deletions and frameshifting intragenic duplication CNVs can lead to allele LOF. Genic CNV can be responsible for 14–60% of disease alleles in selected recent studies of novel disease genes (*BPTF* [59], *NONO* [60], *PSMD12* [61], *TANGO2* [62], *TRIP12* [65]) and 7–26% of families of different disease cohorts (Bardet Biedl ciliopathies [84], primary immune deficiency disorders [85], brain malformations [36], an Arabic DD cohort [86], unsolved clinical exomes [87]). To advance the clinical investigation of CNVs as pathogenic alleles, we studied de novo CNVs and hemizygous deletion CNVs in males, involving both known and candidate disease genes using high-resolution exon-targeted clinical CMA data from over 62,000 patients. Our study indicated four genes *ARGLU1*, *STK3*, *MEIS2*, and *PTCHD1* as having the strongest evidence for disease association.

*ARGLU1* (arginine and glutamate rich protein 1) was reported to play a regulatory role in gene transcription through its interaction with MED1 Mediator complexes [88]*.* The highest expression level of this gene was found in the cerebellum (GTEx database). The neighboring *EFNB2* (Ephrin B2) encodes a member of the Eph receptor family. In previous cytogenetic studies, two large 13q de novo deletions (9 Mb and 28 Mb in size) involving both *ARGLU1* and *EFNB2* were reported in patients with mild anorectal malformations and *EFNB2* was proposed as a good candidate disease gene [89]. In support of this notion, studies in mice revealed that 28% of heterozygous *Efnb2* knockout mice presented with mild anorectal malformations [90], closely resembling those observed in patients with *EFNB2*. However, none of our patients with CNV deletions involving *EFNB2* had anorectal malformations. Most patients manifested neurological anomalies including DD confirmed in three out of four individuals. In addition, clinical evaluation of Pt.1 with a de novo 1 Mb deletion revealed developmental regression and ASD, whereas features of Pt.3 who carries a LOF point mutation in *ARGLU1* included abnormal movement, cerebellar hypoplasia, and oculomotor apraxia. Given that we did not find additional evidence supporting *EFNB2* as a disease-associated gene (i.e. all validated missense variants in *EFNB2* were inherited from healthy parents), we propose that *ARGLU1* rather than *EFNB2* is a better candidate gene responsible for the neurological anomalies.

*STK3* (also known as *MST2*) encodes serine/threonine-protein kinase 3, a component of the Hippo pathway that plays an important role in organ size regulation and tumor suppression by restricting proliferation and promoting apoptosis. Loss of *Hpo* (homologue of *MST1* and *MST2*) in *Drosophila* causes tissue overgrowth [91]. Mouse studies showed that loss of *Mst1* and *Mst2* leads to severe growth retardation and other embryonic abnormalities, suggesting that both genes are crucial in early mouse development [92]. We identified four small-sized deletions encompassing 1–3 exons of the *STK3* gene in individuals with different congenital anomalies (Table 2). Importantly, two of those deletions were de novo events. However, the *STK3* haploinsufficiency score of 0.12 and pLI score of 0 do not support its pathogenicity. The contradictory findings between haploinsufficiency prediction scores and identification of two de novo exonic deletions, which suggested likely pathogenicity of LOF variants in *STK3*, could potentially be explained by incomplete penetrance or alternative disease contributing mechanisms other than haploinsufficiency.

*MEIS2* is expressed during early fetal brain development in humans [93] and was proposed to contribute to the development of tissues originating from the neural crest, similarly to the mouse orthologue found to be expressed in neural crest cells. Homozygous *Meis2* deficiency in mice results in perturbed development of the craniofacial skeleton and abnormalities in the heart and cranial nerves [74, 94]. Thus far, nine deletion CNVs of this gene were identified in patients with cleft palate (seven individuals), atrial or ventricular septal defect (four individuals), and mild to severe ID (eight individuals) [69, 70, 72]. Recently, a more severe phenotype of ID, cleft palate, and heart defects was associated with de novo frameshift deletion (p.Arg333del) and a de novo stop-gain SNV (p.Ser204*), suggesting that a truncated protein may cause a more severe clinical consequence than haploinsufficiency through a potential dominant-negative mechanism [71, 73, 74]. Our patients with a CNV deletion have a relatively milder phenotype, including DD, ASD (three patients), delayed verbal (three patients) and motor milestones (one patient), cleft palate (one patient), and bifid uvula (one patient). In contrast to previously reported cases, none of our patients with CNV deletions had cardiac defects. Similar phenotypic features (asymmetry of the thorax, bifid uvula, and a specific learning disability) were found in one patient with *MEIS2* deletion reported in the DECIPHER database. *MEIS2* maps to 15q14 distal to the Angelman/Prader-Willi syndromes genomic region on 15q11.2q12 and the *CHRNA7* gene on 15q13.3 (located 5 Mb from *MEIS2*). Thus, better understanding of the *MEIS2* alternations may help to elucidate the phenotypic spectrum of patients with larger-sized deletions encompassing proximal chromosome 15q*.*

*PTCHD1* on Xp22.11 is highly expressed in the brain, especially in the cerebellum [80]. Recent functional studies reported that during early postnatal mouse development, *Ptchd1* is selectively expressed in the thalamic reticular nucleus (TRN), a group of GABAergic neurons that regulate thalamo-cortical transmission, sleep rhythms, and attention [95]. It was suggested that *Ptchd1* plays a role in the hedgehog signaling pathway [75]. Moreover, it was shown that a conditional TRN *Ptchd1* deletion causes attention deficit and hyperactivity, whereas the constitutional deletion of this gene leads to a potentially more severe phenotype, including learning impairment, hyper-aggression, and motor defects [95]. Deletions involving *PTCHD1*, the upstream regulatory region encoding *PTCHD1-AS* and *DDX53*, or both were initially reported in individuals with ASD [75–77], ID [78], or both. Identification of additional male patients with CNV deletions or truncating SNVs, involving *PTCHD1*, further support LOF of this gene as disease-contributing for non-syndromic neurodevelopmental disorders, including ID, ASD, hypotonia, and behavioral abnormalities [79, 80]. Importantly, *PTCHD1* is listed in the SFARI gene database (https://gene.sfari.org) as a strong candidate gene for ASD and DD/ID. Consistent with this, we identified three male patients with deletions of *PTCHD1* who all manifested ASD, DD, or both (Table 4).

In summary, we applied computational scores that consider the vulnerability of genes to LOF and variant burden. We found that genes with pathogenic variants based on de novo occurrence also tend to have extreme values of haploinsufficiency scores and variant damage or pathogenicity burden. However, there were some genes that appear to harbor pathogenic variants but had prediction scores arguing against their pathogenicity due to LOF. Although the pattern between computational prediction and de novo variants are generally concordant, the lack of fit reinforces that computational predictions alone are perhaps insufficient for interpreting variation in the absence of transmission information and additional functional studies.

## Conclusions

CNVs are a key class of disease causing variation. Our data further document the efficacy of exon-targeted CMA for the detection of genic and exonic CNVs, complementing WES in clinical diagnostics, and its potential for discovery of novel disease genes. Notably, exon-targeted CMA detected several pathogenic heterozygous and homozygous single-exon CNVs missed by clinical WES analyses. Technological advances and decreasing costs of whole-genome sequencing (WGS) may eventually make this approach a method of choice for detection of both SNVs and small CNVs, thus replacing CMA and WES; nevertheless, the clinical utility and implementation of WGS remains stymied by lack of objective studies documenting improved molecular diagnosis in comparison to WES plus CMA [87].

## Additional files

Additional file 1: Supplementary table reporting single gene de novo or hemizygous deletions or intragenic duplications in recently proposed candidate or not yet associated disease genes. (DOCX 39 kb)

Additional file 2: Supplementary table reporting de novo or hemizygous deletions or intragenic duplications encompassing 2–5 genes in recently proposed candidate or not yet associated disease genes. (DOCX 29 kb)

Additional file 3: Supplementary text discussing *AGBL4* and *CSMD1* as potential novel candidate disease genes. (DOCX 40 kb)

Additional file 4: Supplementary table containing clinical information on patients with additional variants in *ARGLU1/EFNB2*. (DOCX 14 kb)

Additional file 5: Supplementary table containing clinical information on patients with *AGBL4* variants. (DOCX 19 kb)

Additional file 6: Supplementary table containing clinical information on patients with *CSMD1* variants. (DOCX 21 kb)

Additional file 7: Supplementary figure presenting CNVs in *AGBL4*, including de novo (red), inherited (blue), and deletions of unknown inheritance (green). (PDF 104 kb)

Additional file 8: Supplementary figure presenting CNVs in *CSMD1*, including de novo (red), inherited (blue), and deletions of unknown inheritance (green). (PPTX 95 kb)

## Abbreviations

AD: Autosomal dominant
AR: Autosomal recessive
ASD: Autism Spectrum Disorders
BAC: Bacterial artificial chromosome
BCM: Baylor College of Medicine
BG: Baylor Genetics
BHCMG: Baylor-Hopkins Center for Mendelian Genomics
CGH: Comparative genomic hybridization
CMA: Chromosomal microarray analysis
CNV: Copy number variant
DD: Developmental delay
ExAC: The Exome Aggregation Consortium
HGSC: Human Genome Sequencing Center
ID: Intellectual disability
LOF: Loss of function
MAF: Minor allele frequency
OMIM: Online Mendelian Inheritance in Man
PCR: Polymerase chain reaction
SFARI: Simons Foundation Autism Research Initiative
SNV: Single nucleotide variant
TRN: Thalamic reticular nucleus
WES: Whole-exome sequencing
WGS: Whole-genome sequencing
XL: X-linked
