# Supplementary material for: Identification of novel candidate disease genes from de novo exonic copy number variants
Source: Genome Med. 2017 Sep 21;9:83. doi: 10.1186/s13073-017-0472-7 (PMC5607840; doi:10.1186/s13073-017-0472-7)
Supplement: Supplementary file 1 — Supplementary table reporting single gene de novo or hemizygous deletions or intragenic duplications in recently proposed candidate or not yet associated disease genes. (DOCX 39 kb) [file 13073_2017_472_MOESM1_ESM.docx]

**Additional File 1.** Single gene *de novo* or hemizygous deletions or intragenic duplications in recently proposed candidate or not yet associated disease genes

| **Gene** | **Chr** | | **# of single gene *de novo* or hemizygous deletions** | **# of single gene *de novo* or hemizygous intragenic duplications** | **# of single gene inherited deletions** | **# of single gene inherited intragenic duplications** | **Total # of single gene deletions** | **Total # of single gene intragenic duplications** |
| --- | --- | --- | --- | --- | --- | --- | --- | --- |
| *CDH4* | 20 | 2 | | 0 | 0 | 0 | 3 | 0 |
| ***CSMD1**** | 8 | 2 | | 0 | 3 | 4 | 13 | 13 |
| *FRG1* | 4 | 2 | | 0 | 6 | 0 | 10 | 0 |
| *MACROD2* | 20 | 2 | | 0 | 10 | 2 | 59 | 3 |
| ***MEIS2**** | 15 | 2 | | 0 | 0 | 0 | 2 | 0 |
| *ABCC1* | 16 | 0 | | 1 | 0 | 0 | 1 | 2 |
| *ADCY2* | 5 | 1 | | 0 | 0 | 0 | 1 | 0 |
| *CDH18* | 5 | 1 | | 0 | 3 | 0 | 6 | 13 |
| *CDKAL1* | 6 | 1 | | 0 | 2 | 0 | 5 | 0 |
| *CNTN6* | 3 | 0 | | 1 | 10 | 2 | 22 | 10 |
| *CNTNAP4* | 16 | 0 | | 1 | 0 | 0 | 3 | 1 |
| *CPED1* | 7 | 0 | | 1 | 1 | 0 | 3 | 1 |
| *CRK* | 17 | 0 | | 1 | 0 | 0 | 0 | 1 |
| *CSMD3* | 8 | 0 | | 1 | 1 | 0 | 6 | 3 |
| *DIP2C* | 10 | 1 | | 0 | 0 | 1 | 1 | 6 |
| *DLG2* | 11 | 1 | | 0 | 4 | 1 | 20 | 2 |
| *DOCK4* | 7 | 1 | | 0 | 1 | 0 | 3 | 0 |
| *DSEL* | 18 | 1 | | 0 | 2 | 0 | 4 | 0 |
| *FAM174A* | 5 | 1 | | 0 | 0 | 0 | 2 | 0 |
| *FZD1* | 7 | 1 | | 0 | 0 | 0 | 1 | 0 |
| *HNRNPC* | 14 | 1 | | 0 | 0 | 0 | 1 | 0 |
| *KIAA0825* | 5 | 1 | | 0 | 0 | 0 | 6 | 0 |
| *PIK3C3* | 18 | 1 | | 0 | 0 | 0 | 2 | 0 |
| *PRKG2* | 4 | 1 | | 0 | 0 | 0 | 1 | 0 |
| *PTPRT* | 20 | 1 | | 0 | 2 | 0 | 6 | 0 |
| *RALGAPA1* | 14 | 1 | | 0 | 1 | 0 | 2 | 0 |
| *ROBO1* | 3 | 1 | | 0 | 0 | 1 | 4 | 2 |
| *SLC17A6* | 11 | 0 | | 1 | 0 | 1 | 0 | 4 |
| *SLITRK5* | 13 | 1 | | 0 | 1 | 0 | 2 | 0 |
| *SSBP3* | 1 | 1 | | 0 | 0 | 0 | 1 | 0 |
| *STARD13* | 13 | 0 | | 1 | 0 | 0 | 0 | 1 |
| *STC1* | 8 | 1 | | 0 | 0 | 0 | 1 | 0 |
| ***STK3**** | 8 | 1 | | 0 | 0 | 0 | 3 | 0 |
| *STPG2* | 4 | 0 | | 1 | 1 | 0 | 7 | 1 |
| *TANC2* | 17 | 1 | | 0 | 0 | 0 | 1 | 0 |
| *TFEB* | 6 | 0 | | 1 | 0 | 0 | 0 | 1 |
| *TRAPPC10* | 21 | 1 | | 0 | 0 | 0 | 1 | 0 |
| *UBN1* | 16 | 1 | | 0 | 0 | 0 | 1 | 0 |
| *VIPR2* | 7 | 1 | | 0 | 0 | 0 | 2 | 1 |
| *ZMIZ1* | 10 | 1 | | 0 | 0 | 0 | 1 | 0 |
| *ZMYM2* | 13 | 1 | | 0 | 0 | 0 | 1 | 0 |
| *ZNHIT6* | 1 | 1 | | 0 | 0 | 0 | 1 | 0 |
| *MAP7D3* | X | 20 | | 0 | 3 | 0 | 20 | 0 |
| *VAMP7* | X | 8 | | 0 | 3 | 0 | 8 | 0 |
| *EDA2R* | X | 6 | | 0 | 3 | 0 | 6 | 0 |
| *DCAF12L2* | X | 4 | | 0 | 1 | 0 | 4 | 0 |
| *DDX53* | X | 4 | | 0 | 1 | 0 | 3 | 0 |
| ***PTCHD1**** | X | 4 | | 0 | 2 | 0 | 4 | 0 |
| *CPXCR1* | X | 3 | | 0 | 1 | 0 | 3 | 0 |
| *GPR64* | X | 3 | | 0 | 1 | 0 | 3 | 0 |
| *CLCN4* | X | 2 | | 0 | 1 | 0 | 2 | 0 |
| *DACH2* | X | 2 | | 0 | 0 | 0 | 2 | 0 |
| *GPR112* | X | 2 | | 0 | 0 | 0 | 2 | 0 |
| *HS6ST2* | X | 2 | | 0 | 0 | 0 | 2 | 0 |
| *MAGEC3* | X | 2 | | 0 | 0 | 0 | 2 | 0 |
| *PCDH11X* | X | 2 | | 0 | 0 | 0 | 2 | 0 |
| *PGRMC1* | X | 2 | | 0 | 0 | 0 | 2 | 0 |
| *ACTRT1* | X | 1 | | 0 | 0 | 0 | 1 | 0 |
| *APOOL* | X | 1 | | 0 | 0 | 0 | 1 | 0 |
| *ARSD* | X | 1 | | 0 | 1 | 0 | 1 | 0 |
| *ARSF* | X | 1 | | 0 | 0 | 0 | 1 | 0 |
| *ASB11* | X | 1 | | 0 | 0 | 0 | 1 | 0 |
| *AWAT1* | X | 1 | | 0 | 0 | 0 | 1 | 0 |
| *BRCC3* | X | 1 | | 0 | 0 | 0 | 1 | 0 |
| *CD99L2* | X | 1 | | 0 | 0 | 0 | 1 | 0 |
| *CRLF2* | X | 1 | | 0 | 0 | 0 | 1 | 0 |
| *EFHC2* | X | 0 | | 1 | 0 | 0 | 0 | 0 |
| *EGFL6* | X | 1 | | 0 | 0 | 0 | 1 | 0 |
| *ENOX2* | X | 1 | | 0 | 0 | 0 | 1 | 0 |
| *FIGF* | X | 1 | | 0 | 1 | 0 | 1 | 0 |
| *GABRQ* | X | 1 | | 0 | 0 | 0 | 1 | 0 |
| *GLOD5* | X | 1 | | 0 | 0 | 0 | 1 | 0 |
| *GLRA2* | X | 1 | | 0 | 0 | 0 | 1 | 0 |
| *GPM6B* | X | 1 | | 0 | 0 | 0 | 1 | 0 |
| *GUCY2F* | X | 1 | | 0 | 0 | 0 | 1 | 0 |
| *HDHD1* | X | 1 | | 0 | 0 | 0 | 0 | 0 |
| *HTR2C* | X | 1 | | 0 | 1 | 0 | 1 | 0 |
| *KLHL13* | X | 0 | | 1 | 0 | 0 | 0 | 0 |
| *LDOC1* | X | 1 | | 0 | 0 | 0 | 1 | 0 |
| *MAP3K15* | X | 1 | | 0 | 0 | 0 | 1 | 0 |
| *MOSPD1* | X | 1 | | 0 | 0 | 0 | 1 | 0 |
| *PASD1* | X | 1 | | 0 | 0 | 0 | 1 | 0 |
| *RAP2C* | X | 1 | | 0 | 0 | 0 | 1 | 0 |
| *SH3KBP1* | X | 0 | | 1 | 0 | 0 | 0 | 0 |
| *SHROOM2* | X | 1 | | 0 | 0 | 0 | 1 | 0 |
| *TBC1D8B* | X | 1 | | 0 | 0 | 0 | 1 | 0 |
| *TENM1* | X | 1 | | 0 | 0 | 0 | 1 | 0 |
| *TEX13B* | X | 1 | | 0 | 0 | 0 | 1 | 0 |
| *WDR44* | X | 1 | | 0 | 0 | 0 | 1 | 0 |
| *ZNF185* | X | 1 | | 0 | 0 | 0 | 1 | 0 |
| *ZNF75D* | X | 1 | | 0 | 0 | 0 | 1 | 0 |

***** - discussed in this paper
