# Supplementary material for: Identification of novel candidate disease genes from de novo exonic copy number variants
Source: Genome Med. 2017 Sep 21;9:83. doi: 10.1186/s13073-017-0472-7 (PMC5607840; doi:10.1186/s13073-017-0472-7)
Supplement: Supplementary file 2 — Supplementary table reporting de novo or hemizygous deletions or intragenic duplications encompassing 2–5 genes in recently proposed candidate or not yet associated disease genes. (DOCX 29 kb) [file 13073_2017_472_MOESM2_ESM.docx]

**Additional File 2.** *De novo* or hemizygous deletions or intragenic duplications encompassing 2-5 genes in recently proposed candidate or not yet associated disease genes

| **Gene** | **Chr** | **# of 2-5 gene *de novo* or hemizygous deletions** | **# of 2-5 gene inherited deletions** | **Total # of 2-5 gene deletions** |
| --- | --- | --- | --- | --- |
| *BCR* | 22 | 2 | 4 | 12 |
| *DLL1* | 6 | 2 | 0 | 2 |
| *FAM120B* | 6 | 2 | 0 | 2 |
| *GNAZ* | 22 | 2 | 4 | 12 |
| *IGLL5* | 22 | 2 | 2 | 9 |
| *RAB36* | 22 | 2 | 4 | 12 |
| *RSPH14* | 22 | 2 | 4 | 12 |
| *ABHD17B* | 9 | 1 | 0 | 1 |
| *ACP6* | 1 | 1 | 0 | 1 |
| *ADCY9* | 16 | 1 | 0 | 1 |
| ***AGBL4**** | 1 | 1 | 0 | 2 |
| *ANKMY2* | 7 | 1 | 0 | 1 |
| *ANKS1B* | 12 | 1 | 0 | 1 |
| *ANXA11* | 10 | 1 | 2 | 9 |
| ***ARGLU1**** | 13 | 1 | 0 | 1 |
| *ATP9B* | 18 | 1 | 0 | 1 |
| *BACE2* | 21 | 1 | 0 | 1 |
| *BCL9* | 1 | 1 | 0 | 1 |
| *BEGAIN* | 14 | 1 | 0 | 1 |
| *BOC* | 3 | 1 | 0 | 1 |
| *BPTF* | 17 | 1 | 0 | 1 |
| *BZW2* | 7 | 1 | 0 | 1 |
| *C9orf85* | 9 | 1 | 0 | 1 |
| *CASC4* | 15 | 1 | 0 | 1 |
| *CCDC127* | 5 | 1 | 0 | 1 |
| *CELF4* | 18 | 1 | 0 | 2 |
| *CFAP44* | 3 | 1 | 0 | 1 |
| *CHAF1B* | 21 | 1 | 0 | 2 |
| *CHD1L* | 1 | 1 | 0 | 1 |
| *CMYA5* | 5 | 1 | 0 | 3 |
| *CRYZL1* | 21 | 1 | 0 | 1 |
| *CTDSPL2* | 15 | 1 | 0 | 1 |
| *CYP46A1* | 14 | 1 | 0 | 1 |
| *DEGS2* | 14 | 1 | 0 | 1 |
| *DLK1* | 14 | 1 | 0 | 1 |
| *DOPEY2* | 21 | 1 | 0 | 2 |
| *DSCAM* | 21 | 1 | 0 | 1 |
| ***EFNB2**** | 13 | 1 | 0 | 1 |
| *ELAVL4* | 1 | 1 | 0 | 2 |
| *EML1* | 14 | 1 | 0 | 1 |
| *ERICH1* | 8 | 1 | 0 | 5 |
| *EVL* | 14 | 1 | 0 | 1 |
| *FAM71C* | 12 | 1 | 0 | 1 |
| *FAM84B* | 8 | 1 | 0 | 1 |
| *FBXO25* | 8 | 1 | 0 | 6 |
| *FMO5* | 1 | 1 | 0 | 1 |
| *FOCAD* | 9 | 1 | 0 | 2 |
| *FRMD5* | 15 | 1 | 0 | 1 |
| *FTSJ2* | 7 | 1 | 0 | 2 |
| *GPR45* | 2 | 1 | 0 | 1 |
| *HHIPL1* | 14 | 1 | 0 | 1 |
| *HOMER1* | 5 | 1 | 0 | 1 |
| *HRCT1* | 9 | 1 | 0 | 1 |
| *HSPA13* | 21 | 1 | 0 | 1 |
| *ITSN1* | 21 | 1 | 0 | 1 |
| *JMY* | 5 | 1 | 0 | 1 |
| *KIAA1328* | 18 | 1 | 0 | 2 |
| *L3MBTL3* | 6 | 1 | 0 | 1 |
| *LOC388813* | 21 | 1 | 0 | 1 |
| *LRRC14B* | 5 | 1 | 2 | 4 |
| *LRRC72* | 7 | 1 | 0 | 1 |
| *MAD1L1* | 7 | 1 | 0 | 3 |
| *MAP3K5* | 6 | 1 | 0 | 1 |
| *MAP7* | 6 | 1 | 0 | 1 |
| *MLLT3* | 9 | 1 | 0 | 2 |
| *MORC3* | 21 | 1 | 0 | 2 |
| *MPHOSPH8* | 13 | 1 | 0 | 1 |
| *MRPS9* | 2 | 1 | 0 | 1 |
| *MSRA* | 8 | 1 | 0 | 1 |
| *NFATC1* | 18 | 1 | 0 | 1 |
| *NKD2* | 5 | 1 | 0 | 1 |
| *NOL11* | 17 | 1 | 0 | 1 |
| *NRIP1* | 21 | 1 | 0 | 1 |
| *NUDT1* | 7 | 1 | 0 | 2 |
| *OR2S2* | 9 | 1 | 0 | 1 |
| *OSR2* | 8 | 1 | 0 | 1 |
| *PAPD4* | 5 | 1 | 0 | 3 |
| *PDE1A* | 2 | 1 | 0 | 1 |
| *PDGFA* | 7 | 1 | 0 | 1 |
| *PLAC4* | 21 | 1 | 0 | 1 |
| *PLAC9* | 10 | 1 | 2 | 9 |
| *PLEKHG4B* | 5 | 1 | 2 | 4 |
| *POU3F3* | 2 | 1 | 0 | 1 |
| *POU5F1B* | 8 | 1 | 0 | 1 |
| *PPP1R1C* | 2 | 1 | 0 | 1 |
| *PRKAB2* | 1 | 1 | 0 | 1 |
| *PRKAR1B* | 7 | 1 | 0 | 1 |
| *PSMB1* | 6 | 1 | 0 | 1 |
| *RTL1* | 14 | 1 | 0 | 1 |
| *SALL3* | 18 | 1 | 0 | 1 |
| *SAMD3* | 6 | 1 | 0 | 2 |
| *SAMSN1* | 21 | 1 | 0 | 1 |
| *SFTPD* | 10 | 1 | 1 | 8 |
| *SIDT1* | 3 | 1 | 0 | 1 |
| *SLC12A7* | 5 | 1 | 0 | 1 |
| *SLCO5A1* | 8 | 1 | 0 | 1 |
| *SNX8* | 7 | 1 | 0 | 1 |
| *SPICE1* | 3 | 1 | 0 | 1 |
| *SRL* | 16 | 1 | 0 | 1 |
| *SSFA2* | 2 | 1 | 0 | 1 |
| ***STK3**** | 8 | 1 | 0 | 1 |
| *SULF1* | 8 | 1 | 0 | 1 |
| *TDRP* | 8 | 1 | 0 | 6 |
| *TGFBRAP1* | 2 | 1 | 0 | 1 |
| *TMEM2* | 9 | 1 | 0 | 1 |
| *TMEM254* | 10 | 1 | 2 | 9 |
| *TNKS* | 8 | 1 | 0 | 1 |
| *TPTE2* | 13 | 1 | 0 | 1 |
| *TRIML1* | 4 | 1 | 3 | 16 |
| *TRIML2* | 4 | 1 | 3 | 18 |
| *USP25* | 21 | 1 | 0 | 1 |
| *WDR76* | 15 | 1 | 0 | 1 |
| *ZFP42* | 4 | 1 | 3 | 12 |
| *ZNF596* | 8 | 1 | 0 | 5 |
| *SPRY3* | X | 6 | 0 | 6 |
| *VAMP7* | X | 6 | 0 | 6 |
| *IL9R* | X | 4 | 0 | 4 |
| *BRCC3* | X | 1 | 0 | 1 |
| *CMC4* | X | 1 | 0 | 1 |
| *CXorf67* | X | 1 | 0 | 1 |
| *FAM120C* | X | 1 | 1 | 1 |
| *FUNDC2* | X | 1 | 0 | 1 |
| *GSPT2* | X | 1 | 0 | 1 |
| *IL1RAPL2* | X | 1 | 0 | 1 |
| *MAGED1* | X | 1 | 0 | 1 |
| *MTCP1* | X | 1 | 0 | 1 |
| *NRK* | X | 1 | 0 | 1 |
| *NUDT10* | X | 1 | 0 | 1 |
| *NUDT11* | X | 1 | 0 | 1 |
| *PNPLA4* | X | 1 | 1 | 1 |
| *TMEM255A* | X | 1 | 0 | 1 |
| *VCX2* | X | 1 | 1 | 1 |
| *WNK3* | X | 1 | 1 | 1 |
| *ZBTB33* | X | 1 | 0 | 1 |

***** - discussed in this paper
