# Supplementary material for: Identification of novel candidate disease genes from de novo exonic copy number variants
Source: Genome Med. 2017 Sep 21;9:83. doi: 10.1186/s13073-017-0472-7 (PMC5607840; doi:10.1186/s13073-017-0472-7)
Supplement: Supplementary file 3 — Supplementary text discussing AGBL4 and CSMD1 as potential novel candidate disease genes. (DOCX 40 kb) [file 13073_2017_472_MOESM3_ESM.docx]

**Additional File 3**

***AGBL4***

We have found 17 CNV deletions involving *AGBL4* on 1q33. One of these deletions was confirmed to be *de novo*, two deletions were inherited, and the inheritance of the remaining 14 deletions is unknown (Additional File 5 and Additional File 7). There is one intragenic deletion encompassing exons 3-6 of unknown inheritance identified in a DECIPHER patient 286816 without any clinical information and one maternally inherited deletion encompassing exon 3 found in a DECIPHER patient 256130 without any clinical information.

*AGBL4* is a member of the cytosolic carboxypeptidase (CCP) family that catalyzes the deglutamylation of polyglutamate side chains generated by post-translational polyglutamylation in proteins such as tubulins. Mouse genetic studies revealed that knockout of another gene from the CCP family (*CCP1* also known as *Nna1*) leads to neurodegeneration due to microtubule hyperglutamylation [1]. Recently, it was further confirmed that Nna1, CCP4, and CCP6 (homologs of human AGBL4) all metabolize polyglutamate side chains of tubulin; however, they have distinct kinetic properties and preferences for glutamate chain length and different requirements for acidic amino acids near the branch point glutamate [2]. In particular it was shown that CCP4 and CCP6 are not functionally equivalent to Nna1 *in vivo* [2]. AGBL4 expression in human is enhanced in cerebral cortex and fallopian tube (GTEx database). The important role of *AGBL4* in neurodevelopment could potentially explain phenotypic features observed in patients with CNV deletions. Importantly, in ten out of eighteen individuals some form of developmental delay was reported (Additional File 5). Although *AGBL4* seems to be tolerant to LoF variants (pLI score =0), it may exhibit haploinsufficiency (Haploinsufficiency score of 3.55%). Together with our results this may suggest that *AGBL4* CNV deletions may play role in the susceptibility to neurodevelopmental disorders.

***CSMD1***

We identified nine different-sized CNV deletions involving *CSMD1.* One of these deletions was confirmed to be *de novo*. The contribution of this *de novo* CNV is not clear, since in the same patient a predicted deleterious, *de novo* missense variant was found in *SMC1A*, which likely explain the patient major phenotype (CDLS). Two deletions were paternally inherited, one deletion was maternally inherited, and the inheritance of four other CNVs is unknown (Additional File 6 and Additional File 8). In the DECIPHER database, there are also at least four intragenic, exon-including CNV deletions that were inherited. *CSMD1* were not previously associated with disease, and the prediction scores do not favor haploinsufficiency mechanism *CSMD1* (haploinsufficiency score of *CSMD1* is 47.53). However, the identification of *de novo* deletions suggests potential disease-association or susceptibility.

*CSMD1,* encoding CUB and Sushi multiple domains-1 protein, is highly expressed in cerebellum, substantia nigra, hippocampus, and fetal brain and was found to be a target of mir-137 that regulates adult neurogenesis [3] and neural maturation [4]. CSMD1 is a complement control-related protein suggested to inhibit the canonical complement pathway. It was proposed that loss of CSMD1 function may lead to impaired regulation of the canonical complement cascade or may influence the regulation of synaptic functions [5–7]. *CSMD1* has been previously associated with schizophrenia [8–13]. More recent GWAS and WES studies indicated its potential role also in ASD [14,15]. Mouse studies confirmed a high expression of *Csmd1* in the central nervous system [16]. Neuropsychological deficits including anxiety and depressive endophenotypes were observed in mice with *Csmd1*^-/-^ homozygous knockout alleles [16]*;* no schizophrenia related-behaviors were observed [17]. Using WES, Cukier et al. identified heterozygous damaging missense variants (p. Pro2262Ala and p. Gly827Asp) co-segregating with ASD in two unrelated families [14]. Further query of our CMA database revealed nine other intragenic losses (including one *de novo*) involving *CSMD1,* ranging in size between 24-550 kb. Four patients presented developmental delay or behavior issues (Additional File 6). One of these three deletions encompasses exons 3 and 4 and another is located in the intron between these two exons (Additional File 8). Interestingly, based on the GWAS studies, [9,11,12] the SNP most strongly associated with schizophrenia is located between these two exons. Our findings support the potential disease-contributing or susceptibility role of *CSMD1* in neurodevelopmental disorders.

**References**

1. Rogowski K, van Dijk J, Magiera MM, Bosc C, Deloulme J-C, Bosson A, et al. A family of protein-deglutamylating enzymes associated with neurodegeneration. Cell. 2010;143:564–78.

2. Wu H-Y, Rong Y, Correia K, Min J, Morgan JI. Comparison of the Enzymatic and Functional Properties of Three Cytosolic Carboxypeptidase Family Members. J. Biol. Chem. 2015;290:1222–32.

3. Szulwach KE, Li X, Smrt RD, Li Y, Luo Y, Lin L, et al. Cross talk between microRNA and epigenetic regulation in adult neurogenesis. J. Cell Biol. 2010;189:127–41.

4. Smrt RD, Szulwach KE, Pfeiffer RL, Li X, Guo W, Pathania M, et al. MicroRNA miR-137 regulates neuronal maturation by targeting ubiquitin ligase mind bomb-1. Stem Cells Dayt. Ohio. 2010;28:1060–70.

5. Gunnersen JM, Kim MH, Fuller SJ, De Silva M, Britto JM, Hammond VE, et al. Sez-6 proteins affect dendritic arborization patterns and excitability of cortical pyramidal neurons. Neuron. 2007;56:621–39.

6. Gendrel M, Rapti G, Richmond JE, Bessereau J-L. A secreted complement-control-related protein ensures acetylcholine receptor clustering. Nature. 2009;461:992–6.

7. Xu W, Cohen-Woods S, Chen Q, Noor A, Knight J, Hosang G, et al. Genome-wide association study of bipolar disorder in Canadian and UK populations corroborates disease loci including *SYNE1* and *CSMD1*. BMC Med. Genet. 2014;15:2.

8. Håvik B, Le Hellard S, Rietschel M, Lybæk H, Djurovic S, Mattheisen M, et al. The complement control-related genes *CSMD1* and *CSMD2* associate to schizophrenia. Biol. Psychiatry. 2011;70:35–42.

9. The Schizophrenia Psychiatric Genome-Wide Association Study (GWAS) Consortium. Genome-wide association study identifies five new schizophrenia loci. Nat. Genet. 2011;43:969–76.

10. Kwon E, Wang W, Tsai L-H. Validation of schizophrenia-associated genes *CSMD1, C10orf26, CACNA1C* and *TCF4* as miR-137 targets. Mol. Psychiatry. 2013;18:11–2.

11. Donohoe G, Walters J, Hargreaves A, Rose EJ, Morris DW, Fahey C, et al. Neuropsychological effects of the *CSMD1* genome-wide associated schizophrenia risk variant rs10503253. Genes Brain Behav. 2013;12:203–9.

12. Rose EJ, Morris DW, Hargreaves A, Fahey C, Greene C, Garavan H, et al. Neural effects of the *CSMD1* genome-wide associated schizophrenia risk variant rs10503253. Am. J. Med. Genet. Part B Neuropsychiatr. Genet. Off. Publ. Int. Soc. Psychiatr. Genet. 2013;162B:530–7.

13. Sakamoto S, Takaki M, Okahisa Y, Mizuki Y, Inagaki M, Ujike H, et al. Individual risk alleles of susceptibility to schizophrenia are associated with poor clinical and social outcomes. J. Hum. Genet. 2015;

14. Cukier HN, Dueker ND, Slifer SH, Lee JM, Whitehead PL, Lalanne E, et al. Exome sequencing of extended families with autism reveals genes shared across neurodevelopmental and neuropsychiatric disorders. Mol. Autism. 2014;5:1.

15. Liu X, Shimada T, Otowa T, Wu Y-Y, Kawamura Y, Tochigi M, et al. Genome-wide Association Study of Autism Spectrum Disorder in the East Asian Populations. Autism Res. Off. J. Int. Soc. Autism Res. 2015;

16. Steen VM, Nepal C, Ersland KM, Holdhus R, Nævdal M, Ratvik SM, et al. Neuropsychological deficits in mice depleted of the schizophrenia susceptibility gene *CSMD1*. PloS One. 2013;8:e79501.

17. Distler MG, Opal MD, Dulawa SC, Palmer AA. Assessment of behaviors modeling aspects of schizophrenia in Csmd1 mutant mice. PloS One. 2012;7:e51235.
