# Supplementary material for: Identification of novel candidate disease genes from de novo exonic copy number variants
Source: Genome Med. 2017 Sep 21;9:83. doi: 10.1186/s13073-017-0472-7 (PMC5607840; doi:10.1186/s13073-017-0472-7)
Supplement: Supplementary file 4 — Supplementary table containing clinical information on patients with additional variants in ARGLU1/EFNB2. (DOCX 14 kb) [file 13073_2017_472_MOESM4_ESM.docx]

**Additional File 4.** ARGLU1 and EFNB2 variants

| Case number | Pt5 | Pt 6 | Pt 7 | Pt 8 | Pt 9 |
| --- | --- | --- | --- | --- | --- |
| Variant | 13: 107212003 C>T; c.350G>A; p.R117Q;  missense | 13: 107148097 T>G; c.498A>C; p.Q166H;  missense | 13: 107147339 G>A; c.503C>T; p.A168V;  missense | 13: 107145594 T>C; c.796A>G; p.T266A;  missense | 13: 107145587 G>A; c.803C>T; p.S268L;  missense |
| Confirmation method | PCR + Sanger | PCR + Sanger | PCR + Sanger | PCR + Sanger | PCR + Sanger |
| Affected genes | *ARGLU1* | *EFNB2* | *EFNB2* | *EFNB2* | *EFNB2* |
| Inheritance | inherited | inherited | inherited | inherited | inherited |
| Parental studies | PCR + Sanger | PCR + Sanger | PCR + Sanger | PCR + Sanger | PCR + Sanger |
| General indication for study | **CNS abnormality (5 patients), developmental delay (3 patients), seizures (3 patients)** | | | | |
