# Supplementary material for: Identification of novel candidate disease genes from de novo exonic copy number variants
Source: Genome Med. 2017 Sep 21;9:83. doi: 10.1186/s13073-017-0472-7 (PMC5607840; doi:10.1186/s13073-017-0472-7)
Supplement: Supplementary file 5 — Supplementary table containing clinical information on patients with AGBL4 variants. (DOCX 19 kb) [file 13073_2017_472_MOESM5_ESM.docx]

**Additional File 5.** AGBL4 variants.

| Case number | Pt1 | Pt2 | Pt3 | Pt4 | Pt5 | Pt6 | Pt7 | Pt8 | Pt9 | Pt10 | Pt11 | Pt12 | Pt13 | Pt14 | Pt15 | Pt16 | Pt17 |
| --- | --- | --- | --- | --- | --- | --- | --- | --- | --- | --- | --- | --- | --- | --- | --- | --- | --- |
| Variant | chr1:49,282,185-  50,688,277  1.4 Mb CNV del | chr1:  49,384,946-  49,671,566 *  287 kb CNV del | chr1:  49,501,543-  50,446,163 *  944 kb CNV del | chr1:  50068913-  50278208  209 kb CNV del | chr1:50,251,479-  50,398,437  147 kb CNV del | chr1:  49,501,543  -  50,446,163 *  945 kb CNV del | chr1:  50,220,825-  50,366,361  146 kb CNV del | chr1:  50,220,825-  50,366,361  146 kb CNV del | chr1:49,606,014-  49,912,886 *  306 kb CNV del | chr1:  49,928,778  -  50,601,607 *  673 kb CNV del | chr1:  50,250,730-  50,405,370  *  155 kb CNV del | chr1:  50,250,730-  50,405,370  *  155 kb CNV del | chr1:  50,250,730-  50,405,370  *  155 kb CNV del | chr1:  50,250,730-  50,405,370  *  155 kb CNV del | chr1:  49,436,480-  49,827,935  *  391 kb CNV del | chr1:  49,944,717-  50,022,294 *  78 kb CNV del | chr1:  47,034,115-  50,651,816  3.6 Mb CNV del |
| Confirmation method | HD array | PCR + Sanger | PCR + Sanger | HD array | HD array | PCR + Sanger | HD array | HD array | PCR + Sanger | PCR + Sanger | PCR + Sanger | N/A | N/A | N/A | PCR + Sanger | PCR + Sanger | N/A |
| Inheritance | *de novo* | pat | unk | unk | unk | unk | mat | unk | unk | unk | unk | unk | unk | unk | unk | unk | unk |
| Parental studies | FISH | FISH | N/A | N/A | N/A | N/A | CMA | N/A | N/A | N/A | N/A | N/A | N/A | N/A | N/A | N/A | N/A |
| Speech delay | Mild | N/A | N/A | N/A | N/A | N/A | N/A | N/A | N/A | N/A | N/A | + | N/A | N/A | N/A | N/A | N/A |
| Delayed Motor Skills | + | N/A | N/A | N/A | N/A | N/A | N/A | N/A | N/A | N/A | N/A | N/A | N/A | N/A | N/A | N/A | N/A |
| Behavioral Issues | + | N/A | N/A | N/A | N/A | N/A | N/A | N/A | N/A | N/A | N/A | + | N/A | N/A | N/A | N/A | N/A |
| Dysmorphic Features | N/A | + | N/A | + | N/A | N/A | N/A | N/A | N/A | N/A | N/A |  | N/A | N/A | N/A | N/A | N/A |
| Developmental Delay | N/A | N/A | + | Moderate | Cognitive delay | N/A | + | + | N/A | + | + | + | N/A | + | + | N/A | N/A |
| Mental Retardation | N/A | N/A | N/A | N/A | N/A | N/A | N/A | N/A | N/A | N/A | + | N/A | N/A | N/A | N/A | N/A | N/A |
| Failure to thrive | N/A | N/A | N/A | + | N/A | N/A | N/A | N/A | N/A | N/A | N/A | N/A | N/A | N/A | N/A | N/A | N/A |
| ASD | N/A | N/A | N/A | + | N/A | + | N/A | N/A | N/A | N/A | + | N/A | N/A | + | N/A | + | N/A |
| Hypotonia | N/A | N/A | N/A | N/A | N/A | N/A | N/A | + | N/A | N/A | N/A | N/A | N/A | N/A | N/A | N/A | N/A |
| Microcephally | N/A | N/A | N/A | N/A | N/A | N/A | N/A | + | N/A | N/A | N/A | N/A | N/A | N/A | N/A | N/A | N/A |
| Epilepsy/Seizures | N/A | N/A | N/A | N/A | N/A | N/A | + | N/A | + | N/A | N/A | + | N/A | N/A | N/A | N/A | N/A |
| Other | Sensitivity to loud sounds, trouble falling asleep | Tremor | N/A | Ambiguous Genitalia | N/A | FAS | N/A | N/A | N/A | N/A | N/A | Sleep difficulty | N/A | N/A | N/A | N/A | N/A |

* - CNV coordinates obtained using PCR and Sanger sequencing of breakpoint junction. Abbreviations: N/A – not available; HD array – high density CGH array; unk - unknown
