# Supplementary material for: Identification of novel candidate disease genes from de novo exonic copy number variants
Source: Genome Med. 2017 Sep 21;9:83. doi: 10.1186/s13073-017-0472-7 (PMC5607840; doi:10.1186/s13073-017-0472-7)
Supplement: Supplementary file 6 — Supplementary table containing clinical information on patients with CSMD1 variants. (DOCX 21 kb) [file 13073_2017_472_MOESM6_ESM.docx]

**Additional File 6.** *CSMD1* variants.

| Case number | Pt1 | Pt2 | Pt3 | Pt4 | Pt5 | Pt6 | Pt7 | Pt8 | Pt9 |
| --- | --- | --- | --- | --- | --- | --- | --- | --- | --- |
| Variant | chr8:4,232,020-4,287,861  55 kb, exon 3 CNV del | chr8:2,646,965-3,228,228 *  580 kb, exons 19-70 CNV del | chr8:3,001,841-3,216,490 *  214 kb, exons 22-40 CNV del | chr8:3,832,830-4,391,617 *  558 kb, exons 3-5 CNV del | chr8:3,066,965-3,647,379  580 kb, exons 6-30 CNV del | chr8:3,128,806-3,196,713 *  68 kb, exons 24-26 CNV del | chr8:2,885,653-3,273,571 *  388 kb, exons 13-51 CNV del | chr8:3,141,292-3,184,031  43 kb, exons 24-27 CNV del | chr8:2,999,931-3,022,135  22 kb, exons 38-41 CNV del |
| Confirmation method | N/A | PCR + Sanger | PCR + Sanger | PCR + Sanger | FISH | PCR + Sanger | PCR + Sanger | N/A | N/A |
| Inheritance | *de novo* | pat | mat | unk | pat | unk | unk | unk | unk |
| Parental studies | CMA | FISH | FISH | N/A | FISH | N/A | N/A | N/A | N/A |
| Developmental Delay | N/A | + | + | N/A | N/A | N/A | N/A | N/A | + |
| ADHD | N/A | N/A | N/A | + | N/A | N/A | N/A | N/A | N/A |
| Behavioral issues | N/A | N/A | N/A | + | N/A | N/A | N/A | N/A | N/A |
| Seizures | + | N/A | N/A | N/A | N/A | N/A | N/A | N/A | N/A |
| Microcephaly | + | N/A | N/A | N/A | N/A | N/A | N/A | N/A | N/A |
| Speech Delay | + | N/A | N/A | N/A | N/A | + | + | N/A | N/A |
| ASD/ Autistic Features | + | N/A | N/A | N/A | N/A | + | N/A | N/A | + |
| Dysmorphic Features | mild synophrys, hypotelorism, and hyperpigmentation of the left sclera and adjacent facial skin | N/A | N/A | N/A | N/A | N/A | N/A | N/A | N/A |
| Short Stature | N/A | N/A | + | N/A | N/A | N/A | N/A | N/A | N/A |
| Failure to Thrive | N/A | N/A | + | N/A | N/A | N/A | N/A | N/A | N/A |
| Tetralogy of Falot | N/A | N/A | N/A | N/A | N/A | N/A | N/A | + | N/A |
| Other | N/A | N/A | Asthma, Auricular pit with fistula | N/A | tracheoesophageal fistula, sepsis | Poor balance | Obesity | N/A | N/A |

* - CNV coordinates obtained using PCR and Sanger sequencing of breakpoint junction. Abbreviations: unk – uknown; pat- paternal;

mat – maternal, ASD – Autism Spectrum Disorders, DD – developmental delay; ADHD – attention deficit hyperactivity disorder
