# Supplementary figures and images for: Identification of novel candidate disease genes from de novo exonic copy number variants
Source: Genome Med. 2017 Sep 21;9:83. doi: 10.1186/s13073-017-0472-7 (PMC5607840; doi:10.1186/s13073-017-0472-7)

Additional File 7

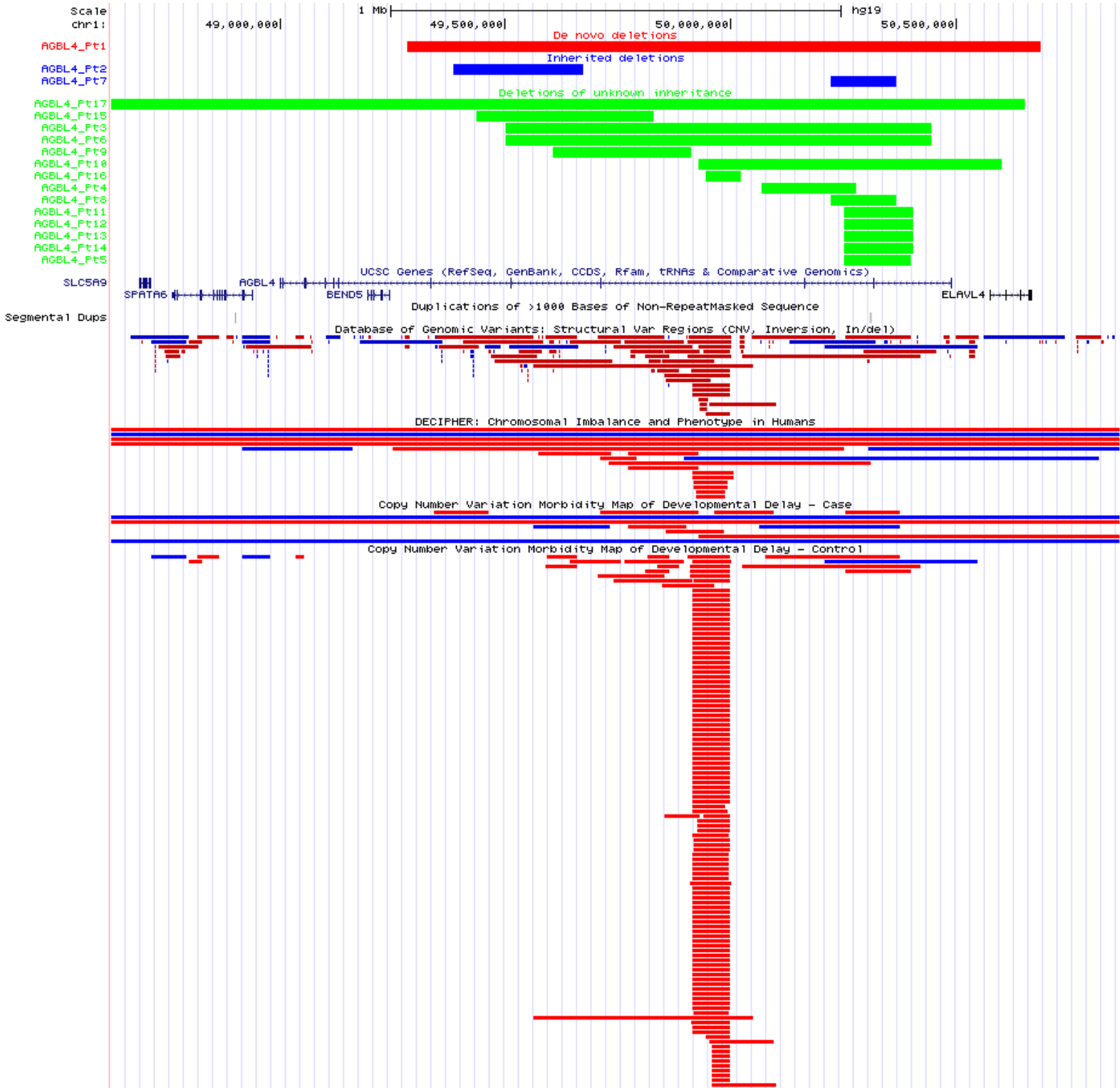

Supplement: Supplementary file 7 — Supplementary figure presenting CNVs in AGBL4, including de novo (red), inherited (blue), and deletions of unknown inheritance (green). (PDF 104 kb) [file 13073_2017_472_MOESM7_ESM.pdf]

## Slide 1
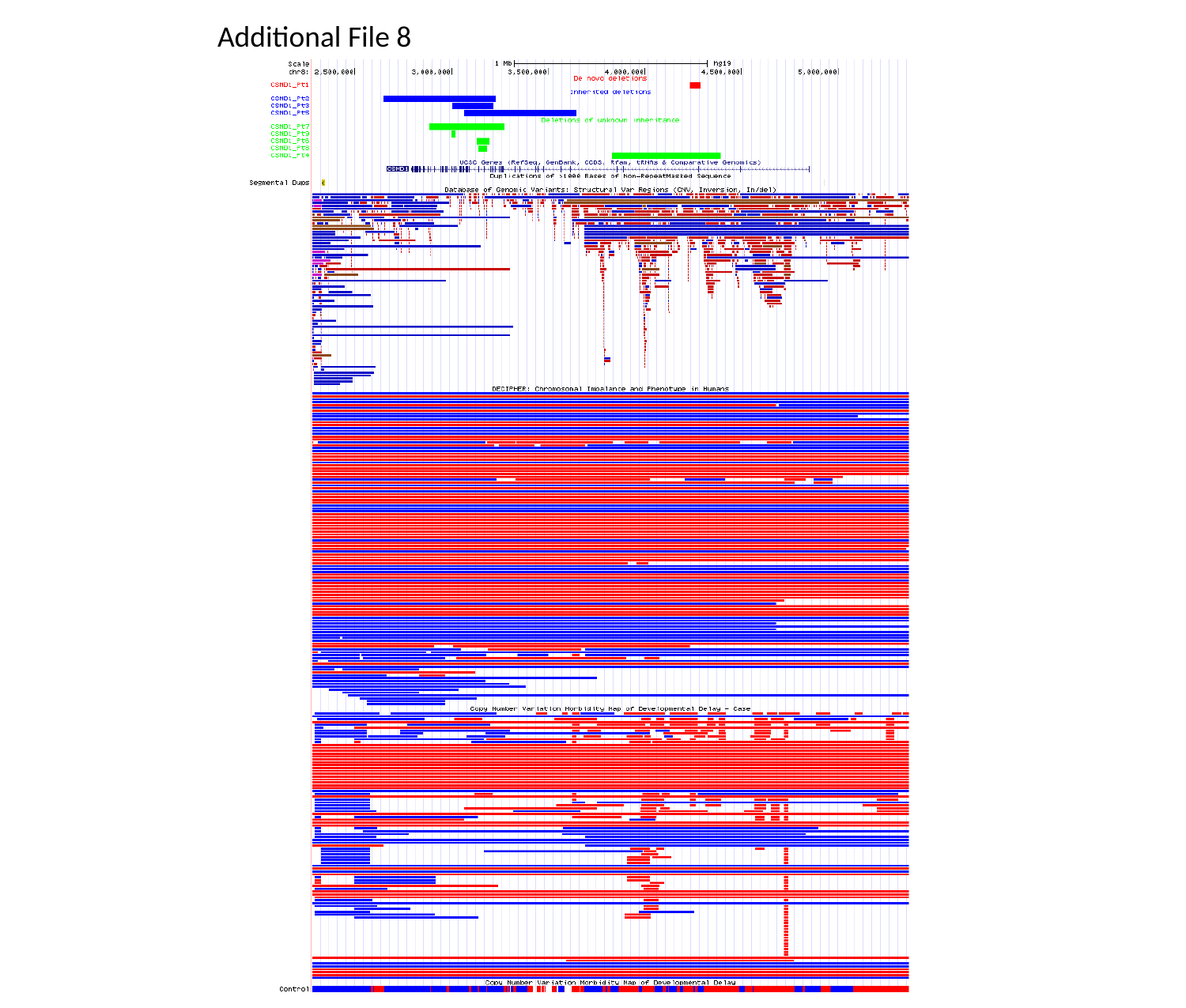

Additional File 8

Supplement: Supplementary file 8 — Supplementary figure presenting CNVs in CSMD1, including de novo (red), inherited (blue), and deletions of unknown inheritance (green). (PPTX 95 kb) [file 13073_2017_472_MOESM8_ESM.pptx]
